# Evaluation of Craniofacial Growth Patterns and Their Association With Airway Obstruction in an Orthodontic Population at a Tertiary Care Hospital in Odisha, India

**DOI:** 10.7759/cureus.85417

**Published:** 2025-06-05

**Authors:** Ipsita Roy, Sanghamitra Jena, Sukriti Mukherjee, Debanwita Dutta, Nivedita Sahoo

**Affiliations:** 1 Orthodontics and Dentofacial Orthopaedics, Kalinga Institute of Dental Sciences, Bhubaneswar, IND; 2 Oral Medicine and Radiology, Kalinga Institute of Dental Sciences, Bhubaneswar, IND; 3 Prosthodontics and Crown and Bridge, Kalinga Institute of Dental Sciences, Bhubaneswar, IND

**Keywords:** airway obstruction, cephalometry, craniofacial growth, orthodontics, population study, vertical growth pattern

## Abstract

Introduction

Airway obstruction is increasingly recognized as a concern in orthodontics due to its potential impact on craniofacial development and overall health. Certain craniofacial growth patterns may predispose individuals to airway obstruction, particularly in populations with vertical growth tendencies. This study aimed to assess the prevalence of airway obstruction in an orthodontic population and explore its association with craniofacial growth patterns.

Methods

A cross-sectional study was conducted using lateral cephalometric radiographs of 225 orthodontic patients (90 males, 135 females) aged 18-30 years who sought treatment at Kalinga Institute of Dental Sciences, a tertiary care hospital in Odisha, India. Radiographs were analyzed to measure six angular craniofacial parameters and nine airway dimensions. Statistical analyses, including t-tests and chi-square tests, were used to assess associations between craniofacial growth patterns and airway dimensions, with a significance level of p ≤ 0.05.

Results

In particular, 42.2% of the analyzed sample exhibited airway obstruction. Vertical growth patterns showed a significant association with airway obstruction (p < 0.001), with a prevalence rate of 72.7% among those with vertical growth patterns. Structural parameters such as reduced airway width, increased hyoid-mandibular plane distance, and elevated mandibular plane angles were linked to increased airflow resistance, suggesting that vertical dysplasia predisposes individuals to narrower airways.

Conclusions

Craniofacial growth patterns, especially vertical dysplasia, significantly impact airway dimensions, highlighting the importance of airway-focused evaluations in orthodontic practice. Targeted interventions, such as mandibular advancement and maxillary expansion, may improve airway patency and enhance overall patient outcomes. These findings underscore the value of early identification and preventive approaches in managing airway health within orthodontic populations.

## Introduction

“Airway-focused orthodontics” refers to a clinical approach that integrates orthodontic interventions not only to achieve dental alignment but also to optimize upper airway function and support overall respiratory health [1]. This has growing clinical relevance as a restricted airway can contribute to conditions such as obstructive sleep apnea (OSA), snoring, and chronic mouth breathing, which may compromise quality of life and systemic health [2].

Several craniofacial and functional factors, such as clockwise mandibular rotation, altered tongue posture, enlarged adenoids, and habitual mouth breathing, can significantly influence hyoid bone position and upper airway morphology [3]. Notably, vertical craniofacial growth patterns are frequently associated with narrowed airways and increased airway resistance [4]. Interventions, such as rapid maxillary expansion (RME) and mandibular advancement, have shown promise in improving airway volume and function [5], although some orthodontic treatments may inadvertently reduce airway dimensions, emphasizing the need to assess airway implications during planning [6].

Recent advances in three-dimensional imaging offer more comprehensive evaluation of airway structures; however, two-dimensional lateral cephalograms continue to be widely used due to their accessibility and cost-effectiveness [7]. While prior studies have explored craniofacial structure-airway relationships in various populations, most originate from Western or urban cohorts [8,9]. There is limited data on orthodontic patients from Eastern India, particularly those seeking treatment at tertiary care centers. Moreover, few studies have examined these associations using standardized cephalometric parameters in adult orthodontic populations.

To address this gap, the present study was designed to (1) assess the prevalence of airway obstruction in an orthodontic population at a tertiary care hospital in Odisha and (2) analyze the association between craniofacial growth patterns and airway dimensions to identify skeletal risk factors for airway compromise.

By addressing these objectives, this study contributes region-specific insights that are clinically relevant for early diagnosis, preventive care, and the development of targeted orthodontic interventions aimed at improving both craniofacial structure and airway function.

## Materials and methods

Study design and population

A cross-sectional, single-centered, retrospective study was conducted using lateral cephalograms of patients from the East Indian population visiting the Department of Orthodontics and Dentofacial Orthopedics at Kalinga Institute of Dental Sciences, a tertiary care hospital in Odisha, between January 2021 and December 2023. All cephalograms were sourced from the institutional database of the Department of Oral Medicine and Radiology, with linear magnification adjusted to ensure consistency.

Ethical approval and consent

The study was approved by the Institutional Review Board (ref: KIDS/RES/02/2025) and the Institutional Ethical Committee (ref: KIIT/KIMS/IEC/2033/2025) of Kalinga Institute of Dental Sciences. Informed consent was obtained from all patients for the use of their radiographic records for research purposes. All patient data were anonymized prior to analysis to ensure confidentiality and compliance with ethical standards.

Sample size

A total of 225 patients (90 males and 135 females) aged between 18 and 30 years were included in the study. Although the study was retrospective and based on available records, a sample size justification was performed based on the primary variable-upper airway width. Using an expected mean difference of 1.9 mm and a pooled standard deviation of 2.35 mm, a minimum of 144 participants (72 per group) was estimated to be sufficient to achieve 80% power at a 5% significance level. Therefore, the inclusion of 225 participants exceeded this requirement and ensured adequate statistical power for comparative analysis.

Inclusion and exclusion criteria

Lateral cephalograms were included if they belonged to patients who were indicated for orthodontic treatment, had no known craniofacial anomalies, and whose radiographs were of high quality with complete accompanying clinical records. Cephalograms were excluded if the patients had a history of orthognathic surgery, systemic conditions affecting craniofacial growth (such as cleft lip or palate), or if the records were incomplete or the radiographs were of poor quality.

Craniofacial and airway parameters

**Figure 1:**
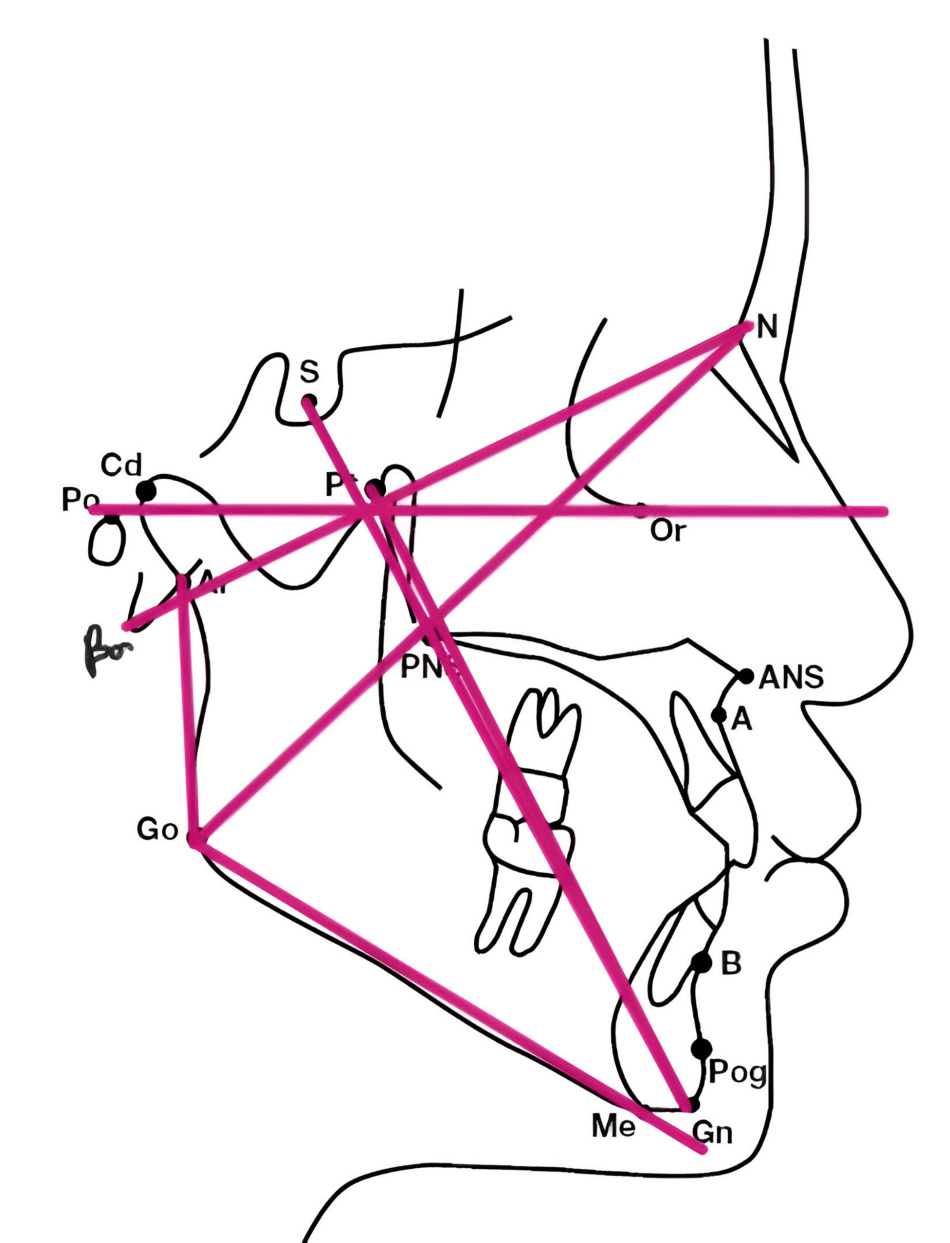
Cephalometric landmarks for growth-related parameters The diagram illustrates key cephalometric landmarks and angular relationships essential for evaluating craniofacial morphology and growth patterns. Notable anatomical points include sella (S), nasion (N), porion (Po), orbitale (Or), posterior nasal spine (PNS), point A, point B, pogonion (Pog), menton (Me), and gonion (Go). The magenta lines denote reference planes such as the cranial base (S–N), Frankfort horizontal plane (Po–Or), and mandibular plane (Go–Me), which are critical for assessing skeletal proportions. The facial axis angle (Ba–N and Ptm–Gn) reflects the direction of facial growth, while the Y-axis (FHP and S–Gn) indicates vertical growth tendency. The gonial angle (Ar–Go–Gn) describes mandibular morphology and is further divided into upper (Ar–Go–N) and lower (N–Go–Gn) components. The mandibular plane angle, formed between Go–Gn and FHP, assesses mandibular inclination relative to the cranial base.

**Figure 2:**
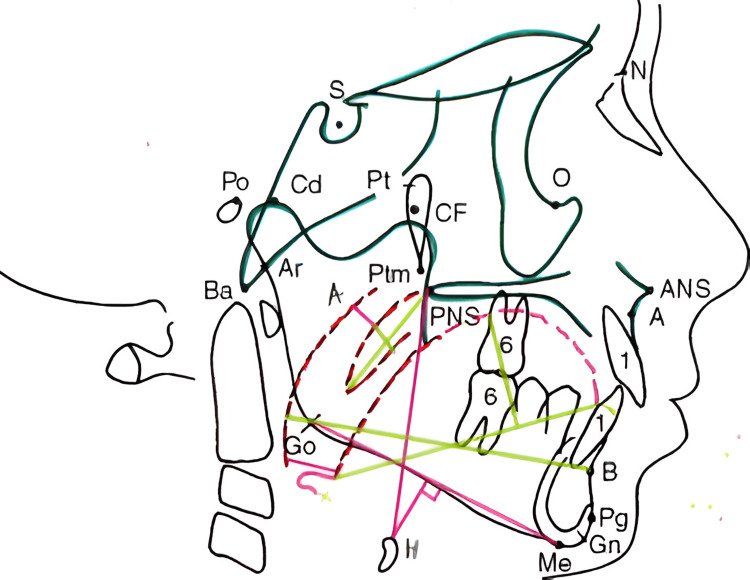
Cephalometric landmarks for airway pattern paratemeters The diagram illustrates key cephalometric landmarks crucial for assessing craniofacial structure, growth direction, and airway dimensions. Notable anatomical points include sella (S), nasion (N), anterior nasal spine (ANS), posterior nasal spine (PNS), point A, point B, basion (Ba), articulare (Ar), porion (Po), condylion (Cd), pterygoid fissure (Pt), pterygomaxillary fissure (Ptm), cranial floor (CF), orbitale (O), gonion (Go), gnathion (Gn), menton (Me), pogonion (Pg), and the hyoid bone (H). Airway parameters are measured using colored reference lines: airway length from PNS to H (magenta), upper and lower airway spaces from adenoid to soft palate and pharyngeal wall to tongue root (magenta), and hyoid–mandibular plane distance from the hyoid to mandibular plane (magenta). Retro-pharyngeal airway space is marked from the pharyngeal wall to B–Go (green). Uvula length and width, tongue length, and tongue height are delineated using green lines along their respective anatomical extents.

Six angular cephalometric parameters were recorded to categorize craniofacial growth patterns, with emphasis on the Y-axis of growth, gonial angle, and mandibular plane angle (Table 1, Figure 1). Airway assessment involved nine measurements, including the airway length, upper and lower airway widths, hyoid mandibular plane (HMP) distance, and soft tissue structures such as uvula length and width (Table 2, Figure 2).

**Table 1:** Cephalometric parameters for the growth pattern This table presents six angular cephalometric parameters used to classify craniofacial growth patterns in the study population. It includes the facial axis angle, Y-axis of growth, gonial angle, upper gonial angle, lower gonial angle, and mandibular plane angle, along with their descriptions and normal ranges. These parameters are essential for distinguishing between horizontal, average, and vertical growth tendencies, providing a foundation for airway-focused orthodontic analysis. *Abbreviations:* Ar: articulare, Ba: basion, FHP: Frankfort horizontal plane, Go: gonion, Gn: gnathion, MP: mandibular plane, N: nasion, Ptm: pterygomaxillary fissure, S: sella

Parameter	Description	Normal range
Facial axis angle	Angle between Ba-N and Ptm-Gn	90 ± 3°
Y-axis of growth	Angle between FHP and S-Gn	59.4 ± 2°
Gonial angle	Angle Ar-Go-Gn	128 ± 7°
Upper gonial angle	Angle Ar-Go-N	71.5 ± 0.9°
Lower gonial angle	Angle N-Go-Gn	55.2 ± 1.1°
Mandibular plane angle	Angle between Go-Gn and FHP	16-28°

**Table 2:** Airway measurement parameters This table defines the nine key measurements used to assess airway dimensions in the study population. Parameters such as airway length, upper and lower airway widths, hyoid mandibular plane (HMP) distance, retro-pharyngeal airway (RPA), uvula length, and tongue dimensions are included, along with their descriptions and normal ranges. These metrics help identify structural deviations associated with airway obstruction. *Abbreviations:* B: basion, Go: gonion, H: hyoid, MP: mandibular plane, PNS: posterior nasal spine

Parameter	Description	Normal range
Airway length	Tangent from PNS to H point	60-70 mm
Upper airway width	Distance between points in upper airway region	15-20 mm
Lower airway width	Distance between points in lower airway region	11-14 mm
Hyoid mandibular plane (HMP)	Perpendicular distance from H point to MP	18.5-23.5 mm
Retro-pharyngeal airway (RPA)	Distance between pharyngeal wall and B-Go	10-11 mm
Uvula length	Soft tissue shadow length of uvula	35 ± 2 mm
Uvula width	Width of uvula	9 ± 1 mm
Tongue length	Distance from tip of tongue to root	84.2 ± 7 mm
Tongue width	Perpendicular drawn from highest tongue shadow	Variable

Measurement reliability

To ensure measurement reliability, intra-observer consistency was evaluated using the intraclass correlation coefficient (ICC) on 20 randomly selected cephalograms. All angular and linear parameters demonstrated ICC values exceeding 0.90, indicating excellent reliability. In addition, the Kappa statistic was used to assess agreement in the classification of craniofacial growth patterns, yielding a value of 0.86, which reflects strong categorical agreement.

Statistical analysis

Data were analyzed using IBM SPSS Statistics for Windows, Version 24.0 (released 2016, IBM Corp., Armonk, NY). Categorical variables were presented as frequencies and percentages, while continuous variables were expressed as means with standard deviations. The normality of continuous variables was assessed using the Shapiro-Wilk test, which confirmed that the data followed a normal distribution. Accordingly, independent samples t-tests were used to compare mean airway measurements between patients with and without airway obstruction. One-way ANOVA, followed by post-hoc tests, was employed, where applicable, to compare continuous variables across multiple craniofacial growth pattern groups. The chi-square test was used to assess the association between craniofacial growth patterns and the presence of airway obstruction. A p-value of less than 0.05 was considered statistically significant.

## Results

Association of growth patterns with airway obstruction

**Figure 3:**
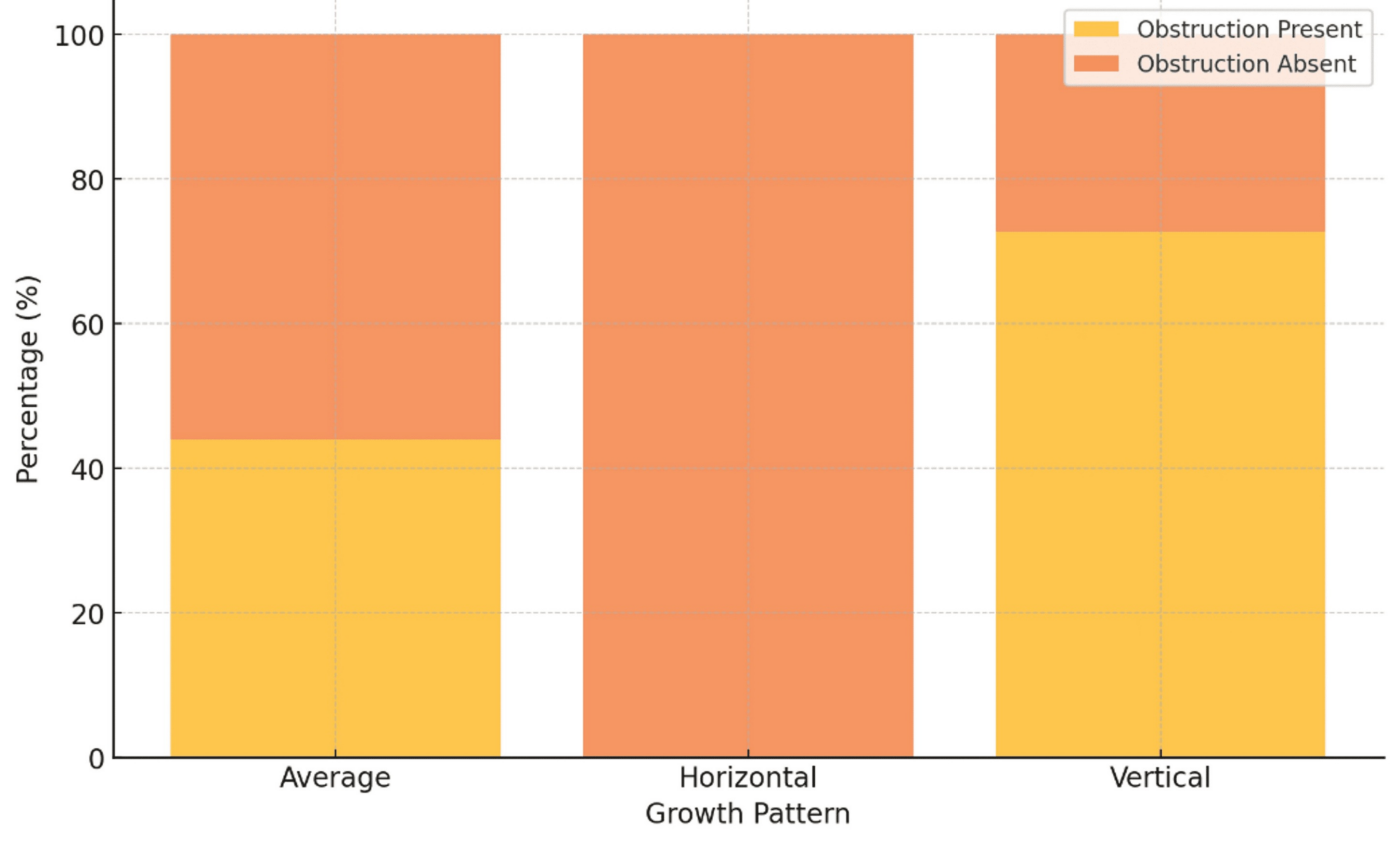
Prevalence of airway obstruction by growth pattern This stacked bar chart illustrates the distribution of airway obstruction across three craniofacial growth patterns (horizontal, average, vertical). Vertical growth patterns show the highest prevalence of obstruction (72.7%), while no cases were observed in the horizontal group, emphasizing the relationship between growth patterns and airway health.

Of the 225 patients analyzed, airway obstruction was observed in 95 individuals (42.2%). The prevalence of airway obstruction was significantly higher in patients with vertical growth patterns (72.7%) compared to those with average growth patterns (44.0%, p < 0.001), while no cases were recorded in individuals with horizontal growth patterns. The chi-square test confirmed a statistically significant association between craniofacial growth patterns and airway obstruction (p < 0.001). These findings suggest that vertical growth patterns, characterized by backward mandibular rotation, predispose individuals to a higher risk of airway obstruction. Conversely, horizontal growth patterns appear to provide a protective influence, with no instances of obstruction observed in this group (Table 3, Figure 3).

**Table 3:** Association of growth patterns with airway obstruction This table shows the frequency of airway obstruction across three craniofacial growth patterns: horizontal, average, and vertical. The table highlights the significant association between vertical growth patterns and airway obstruction, with prevalence and statistical significance (p < 0.001) clearly indicated.

Growth pattern	Airway obstruction (present)	Airway obstruction (absent)	Total sample	p‑value
Average	55 (44.0%)	70 (56.0%)	125	<0.001
Horizontal	0 (0.0%)	45 (100.0%)	45	
Vertical	40 (72.7%)	15 (27.3%)	55	
Total	95 (42.2%)	130 (57.8%)	225	

Comparison of airway dimensions in patients with and without obstruction

Key structural differences in airway dimensions were observed between the obstructed and non-obstructed groups. Independent samples t-tests revealed significantly reduced upper airway width in obstructed patients (9.3 ± 2.3 mm vs. 11.2 ± 2.4 mm, p < 0.001) and lower airway width (8.6 ± 2.6 mm vs. 9.9 ± 2.6 mm, p < 0.001). These findings suggest that pharyngeal narrowing is a major contributor to compromised airway patency in this group. Similarly, the hyoid mandibular plane (HMP) distance was greater in obstructed patients (13.7 ± 6.5 mm) compared to non-obstructed patients (11.7 ± 5.4 mm, p = 0.013), indicating a lower hyoid bone position that further restricts airway dimensions.

**Figure 4:**
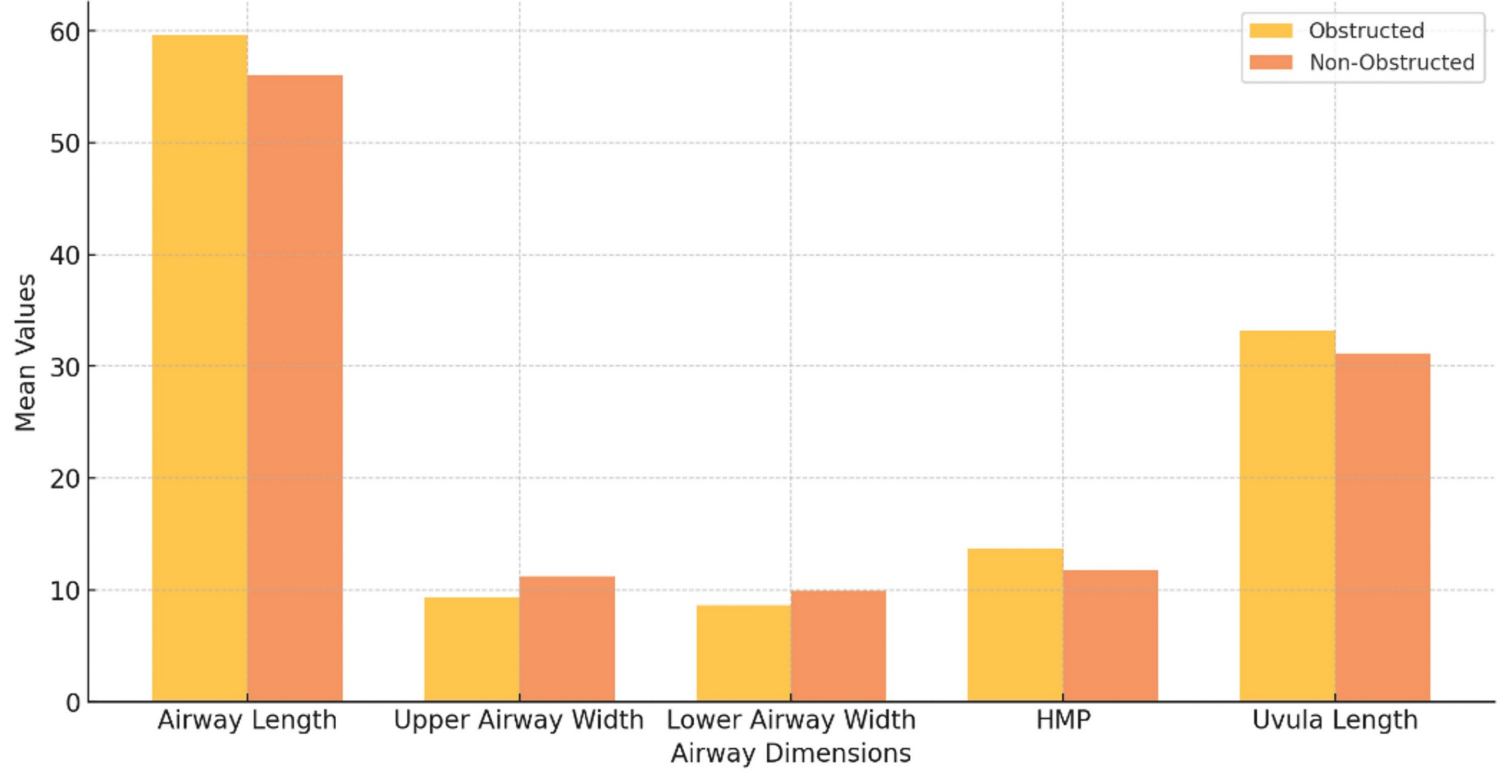
Comparison of significant airway dimensions in patients with airway obstruction This bar chart compares the mean values of significant airway dimensions—such as airway length, upper and lower airway widths, and HMP distance—between the obstructed and non-obstructed groups. The chart highlights specific anatomical measurements that are significantly reduced in obstructed patients, underscoring the role of airway dimensions in obstruction risk. *Abbreviations:* HMP: Hyoid Mandibular Plane.

Uvula length was also significantly increased in the obstructed group (33.2 ± 4.4 mm vs. 31.1 ± 4.1 mm, p < 0.001), which may exacerbate airway narrowing during relaxation states such as sleep. Although the retropharyngeal airway (RPA) width did not differ significantly between groups (5.8 ± 2.3 mm vs. 6.6 ± 3.5 mm, p = 0.060), the observed variability may reflect individual differences in soft tissue composition unrelated to craniofacial growth patterns. Similarly, while the tongue width was comparable between groups (p = 0.352), the tongue length showed a slight reduction in the obstructed group (66.2 ± 7.2 mm vs. 68.3 ± 5.9 mm, p = 0.017). These findings collectively highlight structural variations that contribute to airway obstruction (Table 4, Figure 4).

**Table 4:** Airway dimensions in patients with and without airway obstruction This table compares the airway measurements (e.g., airway length, upper/lower airway widths, HMP distance, uvula length) between patients with and without airway obstruction. Significant differences in parameters are noted, emphasizing structural variations that contribute to obstruction. *Abbreviations:* HMP: hyoid mandibular plane, RPA: retro-pharyngeal airway

Parameter	Airway obstruction (present, mean ± SD)	Airway obstruction (absent, mean ± SD)	p-value
Airway length (mm)	59.6 ± 12.4	56.0 ± 6.9	0.006
Airway width (upper, mm)	9.3 ± 2.3	11.2 ± 2.4	<0.001
Airway width (lower, mm)	8.6 ± 2.6	9.9 ± 2.6	<0.001
HMP (mm)	13.7 ± 6.5	11.7 ± 5.4	0.013
RPA (mm)	5.8 ± 2.3	6.6 ± 3.5	0.060
Uvula length (mm)	33.2 ± 4.4	31.1 ± 4.1	<0.001
Uvula width (mm)	7.4 ± 1.7	7.6 ± 1.7	0.271
Tongue length (mm)	66.2 ± 7.2	68.3 ± 5.9	0.017
Tongue width (mm)	32.5 ± 3.8	32.0 ± 3.7	0.352

Mandibular plane angles and airway obstruction

**Figure 5:**
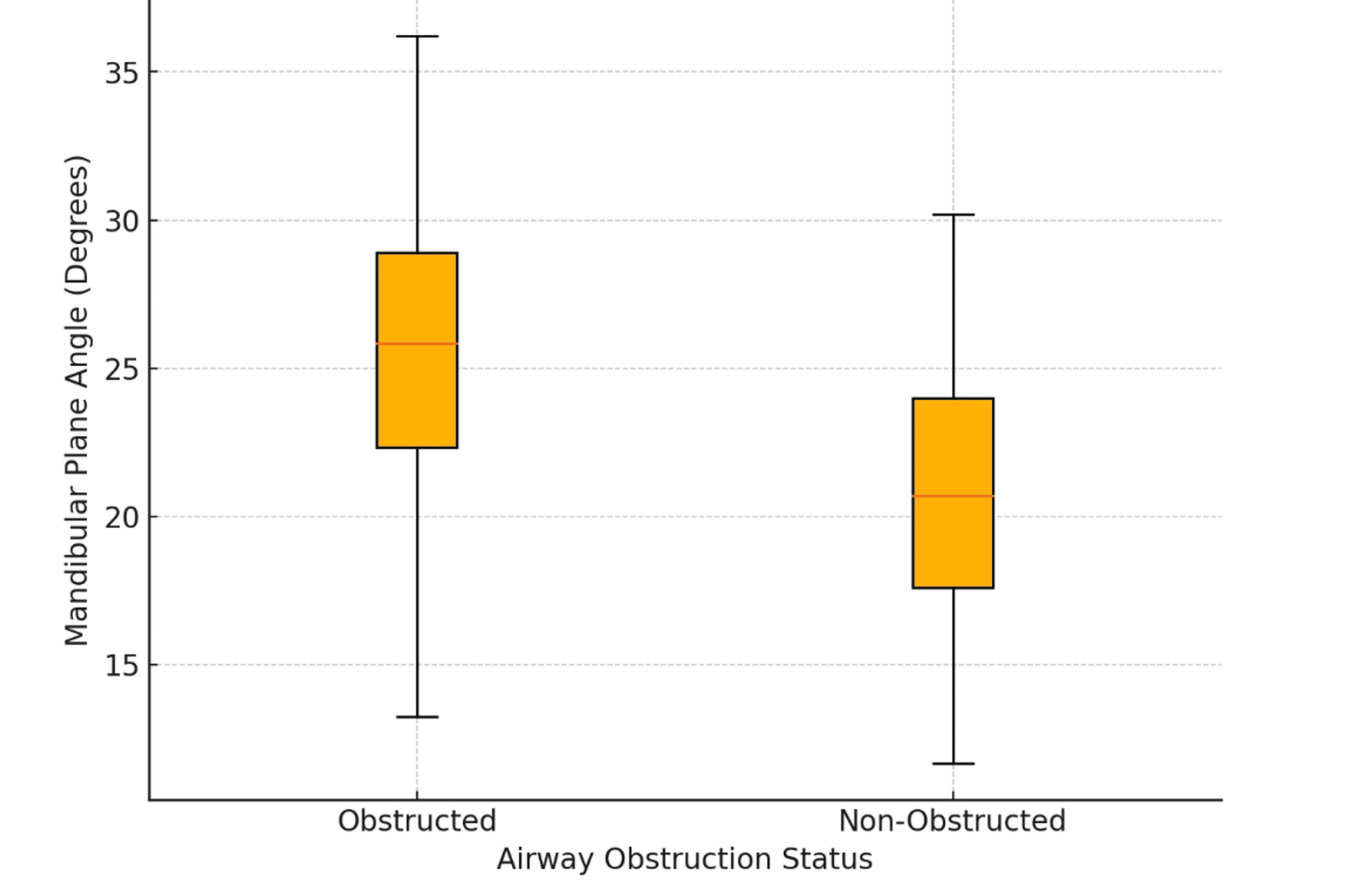
Distribution of mandibular plane angles by airway obstruction status This box plot displays the distribution of mandibular plane angles in patients with and without airway obstruction. The obstructed group exhibits higher median angles, suggesting that backward rotation of the mandible contributes to reduced airway space.

The distribution of mandibular plane angles further supports the link between craniofacial structure and airway patency. Patients with airway obstruction exhibited a higher mean mandibular plane angle (25.4°) compared to non-obstructed patients (20.6°), as determined by an independent samples t-test (p < 0.001). This reflects a backward rotation of the mandible, which repositions the tongue and associated soft tissues posteriorly, reducing airway space. This characteristic backward rotation, prominent in vertical growth patterns, underscores their association with increased airway obstruction, particularly during sleep (Figure 5).

Summary of results

Vertical growth patterns were strongly associated with airway obstruction, primarily due to reduced airway widths, increased HMP distance, and higher mandibular plane angles. These findings emphasize the importance of incorporating airway assessments into routine orthodontic evaluations to identify individuals at risk of airway compromise.

## Discussion

Key findings

This study highlights a significant association between craniofacial growth patterns and airway obstruction, which has critical implications for orthodontic practice and broader healthcare. Vertical growth patterns demonstrated the highest prevalence of airway obstruction (72.7%), while horizontal growth patterns exhibited no cases. These findings underscore the importance of targeted evaluations for at-risk populations and the need for early interventions to address airway health and craniofacial development.

Mechanistic insights

Vertical growth patterns are characterized by backward mandibular rotation, which displaces the tongue and soft tissues posteriorly, leading to airway narrowing. This alteration contributes to clinical symptoms such as snoring, obstructive sleep apnea (OSA), and airflow difficulties during sleep. Reduced upper (9.3 mm vs. 11.2 mm, p < 0.001) and lower airway widths (8.6 mm vs. 9.9 mm, p < 0.001), along with an increased HMP distance (13.7 mm vs. 11.7 mm, p = 0.013), highlight structural factors exacerbating airway restriction. In addition, uvula elongation (33.2 mm vs. 31.1 mm, p < 0.001) further contributes to oropharyngeal narrowing during relaxation states such as sleep.

Comparison with previous studies

The observed prevalence of airway obstruction (42.2%) in this study exceeds the 25% reported by Choudhury et al. [7] in a rural Eastern Indian population. This discrepancy likely reflects the selection of an orthodontic cohort with a higher predisposition to skeletal and craniofacial anomalies. These findings reaffirm the association between vertical dysplasia and reduced airway dimensions, while the lack of significant differences in RPA width (p = 0.060) emphasizes the predominant role of bony structures in this cohort [8-10].

Several studies have investigated airway changes following orthodontic treatment. Pliska et al. [5] evaluated adult patients undergoing extraction and nonextraction treatment protocols and found minimal and clinically insignificant changes in upper airway volume, suggesting that treatment effects on the airway are limited in non-growing individuals and that structural anatomy may be a more dominant factor. By contrast, Alswairki et al. [4], through a meta-analysis, demonstrated that interventions such as rapid maxillary expansion, mandibular advancement, and functional appliances often led to significant increases in airway dimensions, particularly among growing patients. These findings underscore the clinical relevance of airway-focused orthodontics and highlight how treatment effects can vary depending on age, growth pattern, and treatment modality.

Therefore, while the current study emphasizes the role of craniofacial morphology - especially vertical growth patterns - as a predictor of airway obstruction, future longitudinal studies incorporating pre- and post-treatment data would be valuable in assessing the extent to which orthodontic interventions can influence airway patency, especially in at-risk populations.

Clinical implications

The findings emphasize the need for routine incorporation of airway assessments in orthodontic evaluations, particularly for individuals with vertical growth patterns. These assessments can be seamlessly integrated using digital cephalometry and targeted questionnaires to identify risks early. Advanced imaging modalities, such as cone-beam computed tomography (CBCT) and dynamic magnetic resonance imaging (MRI), can further enhance diagnostic accuracy. Early interventions, including rapid maxillary expansion, mandibular advancement, and myofunctional therapy, can improve craniofacial development and airway patency. Multidisciplinary collaboration involving orthodontists, ENT specialists, and sleep medicine professionals is critical for managing complex cases effectively [11-13].

Strengths and limitations

The study’s strengths include its robust sample size and focus on an Eastern Indian population, addressing a significant research gap. However, its cross-sectional design limits causal inference, and reliance on cephalometric measurements may not fully capture dynamic airway behavior. Future longitudinal studies incorporating advanced imaging techniques such as CBCT and MRI could provide deeper insights into airway changes over time. In addition, exploring the impact of early orthodontic interventions on preventing airway disorders could yield valuable strategies for proactive management [14-15].

This study underscores the significant association between craniofacial growth patterns and airway obstruction, particularly in patients with vertical growth patterns, who exhibited a markedly higher prevalence of obstruction. Structural changes, such as reduced upper and lower airway widths, increased Hyoid Mandibular Plane (HMP) distance, and elongated uvula length, were identified as key contributors to compromised airway patency. These findings provide critical insights into the anatomical factors influencing airway health in orthodontic patients.

Despite its strengths, including a robust sample size, the study’s cross-sectional design and reliance on cephalometric measurements call for cautious interpretation. Longitudinal studies with advanced imaging modalities are needed to explore dynamic airway changes and the long-term effects of orthodontic interventions on airway health.

## Conclusions

This study reinforces the evolving role of orthodontics in bridging dental alignment with broader health outcomes, particularly respiratory health. By focusing on an Eastern Indian population, this study adds valuable region-specific data to the limited global literature on airway obstruction and craniofacial morphology. Routine incorporation of airway assessments into orthodontic evaluations, supported by advanced imaging techniques like CBCT and MRI, can enhance early detection of at-risk individuals. Interventions such as rapid maxillary expansion, mandibular advancement, and myofunctional therapy should be prioritized for improving both craniofacial alignment and airway dimensions.

Future advancements in imaging technologies and interdisciplinary approaches hold promise for more effective prevention and management of airway-related complications, ultimately improving patient quality of life.
